# Life worth living or healthy lives? Stakeholder views on biosecurity and animal welfare in EU organic, extensive and small-scale pig and poultry farming systems

**DOI:** 10.1186/s13567-026-01865-0

**Published:** 2026-11-10

**Authors:** Matteo Bassoli, Anne Collin, Jacopo Goracci, Helen Pluschke, Bas Rodenburg, Marina Spinu, Sanna Steenfeldt, Daniela Werner, Anna Zuliani

**Affiliations:** 1 Department of Political Science, Law, and International Studies, University of Padua, Via del Santo, 28, 35123 Padua, Italy; 2 INRAE, Université de Tours, BOA, 37380 Nouzilly, France; 3 Slow Food, Via della Mendicità Istruita, 12042 Bra, Italy; 4 Thünen-Institut Für Ökologischen Landbau, Trenthorst 32, 23847 Westerau, Germany; 5 Faculty of Veterinary Medicine, Utrecht University, Yalelaan 1, 3584 CL Utrecht, The Netherlands; 6 Department of Infectious Diseases and Preventive Medicine, Law and Ethics, University of Agricultural Sciences and Veterinary Medicine, Calea Mănăștur 3-5, 400372 Cluj-Napoca, Romania; 7 Department of Animal and Veterinary Sciences, Aarhus University, Blichers Alle 20, 8830 Tjele, Denmark; 8 Veterinari Senza Frontiere, IZS Delle Venezie, Viale dell’Università, 10, 35020 Legnaro, Italy

**Keywords:** Animal welfare, biosecurity, organic farming, small-scale farming, stakeholder views

## Abstract

Biosecurity is widely recognized as a cornerstone of disease prevention and animal health management. Organic, extensive and small-scale farming systems face several challenges related to biosecurity that are different to more conventional farming settings. This study investigates how farmers, veterinarians and advisors across six European countries view standard biosecurity measures, their effectiveness and their relationship with animal welfare in alternative farms. On the basis of survey data from 243 respondents, we found high awareness of biosecurity but significant concerns about the practicality and suitability of standardized measures in non-intensive settings. Alternative farmers prioritized natural behaviours and outdoor access, whereas veterinarians emphasized disease prevention, revealing different understandings of welfare–health interactions. Consistently, farmers attributed significantly greater importance to welfare dimensions related to natural living, freedom of movement and species-specific behaviours than veterinarians and advisors did. The respondents also expressed concerns about restrictive control measures, including bans on outdoor farming and compulsory culling, which they viewed as potentially undermining their values and the overall farming resilience. These findings highlight the need for policy frameworks that are both scientifically robust and adaptable to diverse farming realities.

**Supplementary information:**

The online version contains supplementary material available at https://doi.org/10.1186/s13567-026-01865-0.

## Introduction

The latest European strategies and policies, such as the Farm-to-Fork Strategy [1] and the Common Agricultural Policy (CAP) [2], aim at fostering the transition towards more sustainable livestock farming systems, with an emphasis on reducing antibiotic use, improving animal welfare and lowering environmental impacts [3]. These objectives also aim to contribute to the Sustainable Development Goals and the One Health agenda, as livestock production practices, in particular those affecting animal comfort and their ability to express innate behaviour, are argued to be synergistic not only with animal and environmental health [3, 4] but also with public health and wellbeing.

In this context, there is growing public interest in alternative or non-intensive farming systems such as sustainable agriculture – organic, extensive and small-scale farming systems – for their potential to address the challenge of sustainable food production [5].

Among others, in the European Union (EU), the pig and poultry sectors play prominent roles in the production of animal-based food [6]. The agricultural census of the EU estimated that 1.63 billion birds were reared across 2.67 million farms. The pig population is estimated to be approximately 134 million heads spread across 2 million farms [7]. Globally, most farms are small and family operated [8]. In the EU, most pigs and poultry are bred in intensive farming systems, but the majority of farms are small scale [7]. While there is a range of definitions of ‘smallness’, most definitions focus on economic size, labour, land managed or the combination of the three [9]. For example, if land managed is used as a unit and 5 ha as a threshold, 60% of EU farms are small scale. Similar results are obtained if economic output is considered as a unit and 8000 Euro/year as a threshold. There is, however, great variability between member states in the number of farms that own or manage less than 5 hectares (e.g. Romania 90%, Italy 60%, France 25%, Belgium 15%, Germany 10% and Denmark 5%) [10]. However, organic farming accounts for approximately 2% of pig and poultry farms in the EU with Denmark, France and Germany representing the largest producers [4, 7]. Figures on outdoor/extensive farms are more blurred, as there is no certification or specification dedicated to outdoor farming, but such systems represent approximately 10–15% of the pig and poultry sector in the EU [11]. The definitory issue is not irrelevant. It is so crucial that it dates back to the seventies [12] and is still contested [13]. This lack of clarity is often systemic and may serve specific economic or policy interests depending on the socio-economic and policy context.

Organic, extensive and small-scale farming systems, while they do not fall under the same definition and may be an extremely heterogeneous group and share important structural and philosophical similarities and founding values (Table 1). These systems typically prioritize lower external inputs, natural animal behaviours and diversified or mixed-use land practices [21, 22], which may also represent shared policy objectives that these farms contribute to achieve and according to which they could be alternatively defined [9, 23].

**Table 1:** **Comparison of farming system types in relation to biosecurity constraints and animal welfare features**

System	Typical characteristics	Welfare features
Organic	Regulated, outdoor access, no routine medications	Emphasis on natural living and low-stress environments [14, 15]
Extensive	Low density, pasture based, mixed use	High behavioural freedom and environmental enrichment [16, 17]
Small scale	Low animal numbers, diverse structures, family owned	Strong human–animal bond, varied husbandry practices [18–20]

While alternative production systems are expected to pay special attention to animals’ behavioural and emotional needs [22], they are often considered in the mainstream literature [24, 25] to be insufficient in terms of disease prevention and management, commonly referred to as biosecurity measures [26]. This mismatch is particularly relevant in the context of trans-boundary animal diseases such as African swine fever (ASF) and avian influenza (AI), where organic, extensive or small-scale pig and poultry farms may be considered by some to be epidemiologically vulnerable and bio-insecure [24, 25] and by others essential for rural resilience and food systems’ transformation [23, 27].

Biosecurity encompasses all practices and physical structures aimed at preventing the introduction and limiting the spread of disease agents within a farm and is considered essential for controlling both endemic and epidemic diseases, thereby ensuring the overall health of animals [26]. Biosecurity policies are often designed primarily to control infectious diseases and protect animal health. The development of biosecurity measures has accompanied the intensification of farming, the progressive confinement of animals and the standardization of inputs and practices, in line with the needs and contexts of intensive farming [28]. Biosecurity involves, among other actions, control over the entry and exit of goods, people and animals; restrictions on the contact of animals with the external environment, especially wildlife; the physical partition of groups; and the cleaning of facilities and disposal of wastes and carcasses [29, 30]. These measures should complement or substitute each other in relation to the characteristics of the farming system [28]. Existing studies often focus on compliance gaps against these standard biosecurity measures [24, 25, 31, 32], suggesting that organic, extensive and small-scale systems face structural challenges in implementing standardized biosecurity measures because of their open layouts, species mixing and resource constraints [24, 33]. Furthermore, in livestock systems characterized by outdoor access, landscape integration and strong human–animal interactions, biosecurity measures may generate trade-offs affecting animal welfare, environmental management and farm resilience [34]. There is, however limited research of socio-cultural and value-based aspects that limit biosecurity implementation in alternative settings within Europe [35–37], especially concerning the relationship between biosecurity measures and animal welfare implications.

Owing to recent developments in the distribution and frequency of severe diseases affecting pigs and poultry [i.e. African swine fever (ASF) and avian influenza (AI)], EU countries are requiring pig and poultry farms to implement even stricter biosecurity measures. Although the law ensuring animal health in the EU [38] encourages the implementation of biosecurity plans, there are no standardized legal requirements, resulting in a wide degree of requirements and practices across EU member states [39, 40].

While animal health is a fundamental aspect of animal welfare [41], the concept of welfare extends beyond the mere absence of disease or other negative states. It also includes the opportunity for animals to experience positive conditions [42] and to live lives worth living [43]. Moreover, engagement in rewarding experiences improves the ability to address a range of challenges and ultimately to maintain or regain healthy physical and mental functioning [42].

Biosecurity is undoubtedly a critical tool for enhancing animal health; however, there is still a limited understanding of how biosecurity measures, as currently framed, are viewed by small-scale, extensive and organic farmers and how these views influence both the adoption of such measures and their perceived impact on animal welfare in these farming systems.

Given these dynamics, we explored the knowledge and values of farmers, veterinarians and advisors regarding animal health, welfare and biosecurity in organic, extensive and small-scale pig and poultry farming systems. This study employed a questionnaire-based approach to shed light on the views of alternative farmers towards standard biosecurity measures and their impact on animal health and welfare. It allowed for a broad, cross-sectoral investigation of the views of different stakeholders working with several animal species and in different countries simultaneously. This in turn helped identify overarching views and broad ideas that can serve as a stepping stone for further research and discussion on biosecurity in alternative farming systems.

By gaining a deeper understanding of how biosecurity measures are viewed, this study contributed to the broader discussion on how biosecurity measures may be adapted to the realities of alternative farming systems.

## Materials and methods

### Study design and sample size

This cross-sectional survey focussed on organic, extensive and small-scale pig and poultry farms and was carried out via an online questionnaire hosted on the Lime Survey platform by the University of Padova, with the link distributed via dedicated mailing lists and direct contact in six European countries participating in the H2020 Poultry and Pig Low-Input and Organic Production Systems’ Welfare (PPILOW) project: France, Germany, Denmark, Romania, the Netherlands, Belgium and Italy. Eligible participants included farmers, veterinarians and advisors aged 18 years and older who were involved in small-scale or extensive or organic farming. Veterinarians and advisors were grouped on the basis of their shared role as external technical experts supporting farm decision-making. With respect to the inclusion and exclusion criteria, we included outdoor certified farms regardless of size and small certified farms regardless of legal status, and we excluded only large indoor non-certified organic farms. Following the Italian and German rules, the thresholds for defining small-scale farming were set at 300 units for pig farming and 3000 units for poultry farming (Table 2). Given the exploratory nature of the research, it was not feasible to create an ex ante sample size. The PPILOW partners decided that a minimum number of 200 replies were needed to obtain results comparable with the relevant literature. The survey opened on 1 May 2024 and was supposed to close on 20 June 2024; then, it was later extended to 1 July 2024. The survey adhered to international ethical standards and followed the Strengthening the Reporting of Observational Studies in Epidemiology (STROBE) guidelines [44]. Formal ethical approval was not required according to the ethical board of the Department of Political Science, Law, and International Studies (University of Padova). Participation was voluntary, and electronic consent was obtained before the respondents completed the questionnaire. The personal data contained in this questionnaire were processed in accordance with the European Union data protection law [45] and in accordance with a policy notice available to participants.

**Table 2:** **Farm type inclusion/exclusion criteria**

Farm	Not certified organic		Certified organic
	Small^*^	Large	Small/large
Outdoor	Included	Included	Included
Indoor	Included	Excluded	NA

^*^Small-scale farms were set at 300 units for pig farming and 3000 units for poultry farming

### Study tools

The questionnaire addressed perceptions of biosecurity measures and their impact on animal health and welfare in the EU; thus, it used questions derived from previous studies focusing on animal welfare or health and biosecurity measures [31, 46–49]. The original version was drafted in English, but it was also disseminated in six national languages: Danish, Dutch, French, German, Italian and Romanian. Following an introductory paragraph explaining the objective of the study and the targeted population and obtaining consent from the respondents, the questionnaire addressed various aspects.

The initial section focussed on ‘animal health and welfare’: it aimed at gauging general knowledge and perceptions via different Likert scales, allowing participants to rate their agreement on a continuum from ‘not important’ to ‘very important’ (see online material for further details). With respect to animal welfare, the participants ranked ten animal welfare criteria by importance [(1) absence of hunger/thirst, (2) comfort around resting, (3) ease of movement, (4) thermal comfort, (5) absence of injuries, (6) absence of diseases, (7) absence of pain, (8) good human–animal relationship, (9) expression of social and other species-specific behaviours, (10) animal positive emotional state] derived from Welfare Quality^®^ [41]. The next section was designed to gather information for evaluating the level of implementation of biosecurity measures (13 items; Appendix A) and the reason for not applying them (8 items). A measure was considered implemented when respondents were replying either ‘always’ or ‘often’. When respondents indicated a low level of implementation (‘never’, ‘rarely’ and ‘sometimes’), they were asked to provide reasons for not implementing the measures. Thereafter, the questionnaire assessed respondents’ concerns regarding the potential impacts of major disease outbreaks, such as African swine fever (ASF) and avian influenza (AI). Participants were asked to identify their primary concerns from a predefined list of consequences, including ‘stamping out’ (culling), the prohibition of outdoor farming, the loss of farm animal genetic lines and the absence of financial compensation. These items were designed to capture the perceived risks and systemic vulnerabilities of alternative farming systems in the face of emergency disease control measures.

In the following section, respondents were asked about biosecurity measures and animal health policies, including their perceived impacts, level of support and perceived adequacy for organic, extensive and small-scale farming system. A pre-final section addressed the respondents’ attitudes: trust (three items), self-efficacy (ten items) and self-esteem (ten items), leaving the final section for farming information (job, farm size, labour units, breeds, type of farming) and demographic and individual characteristics of the participants (age, farming experience, sex, education level, job, geographical setting). The survey is available as supplementary material online. Although the present study is primarily based on quantitative survey data, the interpretation of the results was informed by the interdisciplinary expertise of the research team, encompassing veterinary science, animal welfare, farming practice and policy studies. Open-ended responses were used only to provide contextual insights and were not analysed as a separate qualitative dataset.

### Statistical analysis

The dataset was directly exported from LimeSurvey to SPSS version 29, where all the statistical analyses were performed, assuming a *p*-value of 0.05 for significant results. The main statistical analysis of this study was conducted to explore the underlying structure of variables related to perceptions of biosecurity and animal welfare, as well as to examine the associations between these variables and different professions. With respect to animal welfare, the aforementioned criteria were tested against the three welfare themes described by Lund [21] and Fraser [50]: health and functioning (criteria 1, 4 and 6), natural living (criteria 2, 3, 5 and 7) and affective states (criteria 8, 9 and 10).

To identify latent dimensions underlying participants’ priorities related to animal welfare, we conducted a factor analysis with both orthogonal (Variamax) and oblique rotation (Oblimin). The analysis included those ten welfare variables described in the previous section. Eigenvalues > 0.9 were considered for factor retention, and a maximum of 25 iterations was allowed for factor extraction and rotation. The standard Kaiser‒Meyer‒Olkin (KMO) measure of sampling adequacy and Bartlett’s test of sphericity were used to assess the suitability of the dataset for factor analysis. To investigate the relationships between specific variables, we performed a series of chi-squared tests for independence. The analysis included categorical variables related to participants’ attitudes, behaviours and demographic characteristics. Together, these methods offer a comprehensive statistical exploration of the dataset, addressing both structural and relational aspects of the variables under study.

## Results

### Survey response

A total of 243 full surveys were returned (Table 3), each by an independent respondent from a different farm or professional setting. Farmers represented 58% of the respondents, whereas 33% were veterinarians and advisors. Nine percent of the respondents identified themselves as retired or non-practising veterinarians or advisors. Among pig farmers, certified organic farms represented 56% of the total pig farmers, whereas conventional farms accounted for 33%, of which 25% managed outdoor systems. The remaining 11% were small-scale farmers (<300 pigs) raising their animals conventionally indoors. With respect to poultry, certified organic farms accounted for 60% of the total poultry farms, whereas conventional farms represented 30%, 26% of which were managed outdoors. The remaining 10% were small-scale farmers (<3000 birds) raising their animals conventionally indoors. The median pig farm size was 40 heads (min 2–max 6500), whereas for poultry farmers, the average size was 425 birds (min 10–max 200 000). The largest pig and poultry farms were certified organic. The largest non-organic farms were raising 688 pigs and 90 000 poultry with outdoor access. The participants who took part in the survey had a mean age of 44 years and a mean farming experience of 16 years (Table 4).

**Table 3:** **Country of origin of the respondents and species reared, sorted by country**

Type	Frequency	Percent	Pig	Poultry	Both	Total
Italy	66	27.2	24	26	16	66
Denmark	15	6.2	4	10	1	15
Romania	30	12.3	9	1	20	30
Belgium	55	22.6	18	24	13	55
Germany	12	4.9	0	8	4	12
Netherlands	18	7.4	4	7	7	18
France	45	18.5	18	19	8	45
Other	2	0.8	1	0	1	2
Total	243	100.0	78	95	70	243

**Table 4:** **Size of the pig herd/poultry flock and farmer characteristics**

	Herd size	Flock size	Respondents’ age (years)	Farming experience (years)
Mean	224.73	7269.53	46.33	19.33
Median	40.00	425.00	44.00	16.00
Mode	100.00	200.00	50.00	10.00
Standard deviation	776.53	24528.33	12.50	13.16
Minimum	2.00	10.00	21.00	0.00

### Biosecurity knowledge

A high level of awareness and perceived responsibility regarding biosecurity was reported, with 85% of respondents indicating familiarity with the concept and 87% expressing a sense of responsibility for its implementation. The majority of the respondents claimed to have sufficient information to implement biosecurity measures on their farm or the farms they visited (71% agreed or strongly agreed with the statement), but almost half of the farmers (44%) believed that biosecurity could not be improved on their farm. The only significant difference between the responses of farmers and veterinarians/advisors concerned the perception of whether biosecurity could be improved on their farm, with farmers being more likely to disagree with this statement, indicating a lower perceived potential for further improvement (Appendix B.1).

### Biosecurity implementation

**Figure 1:**
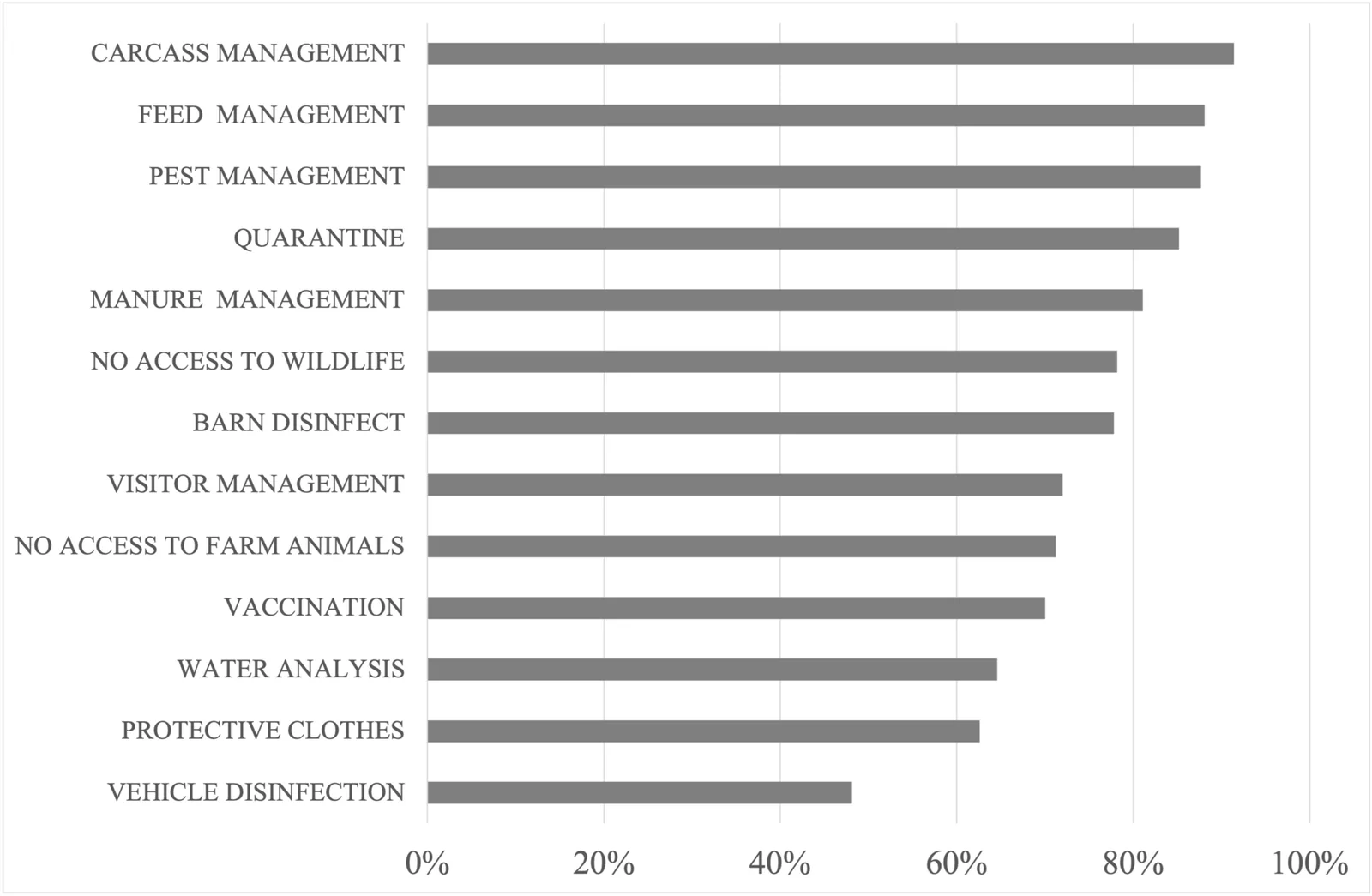
**Frequency of implementation (always/often) of selected biosecurity measures (percentage)**

**Figure 2:**
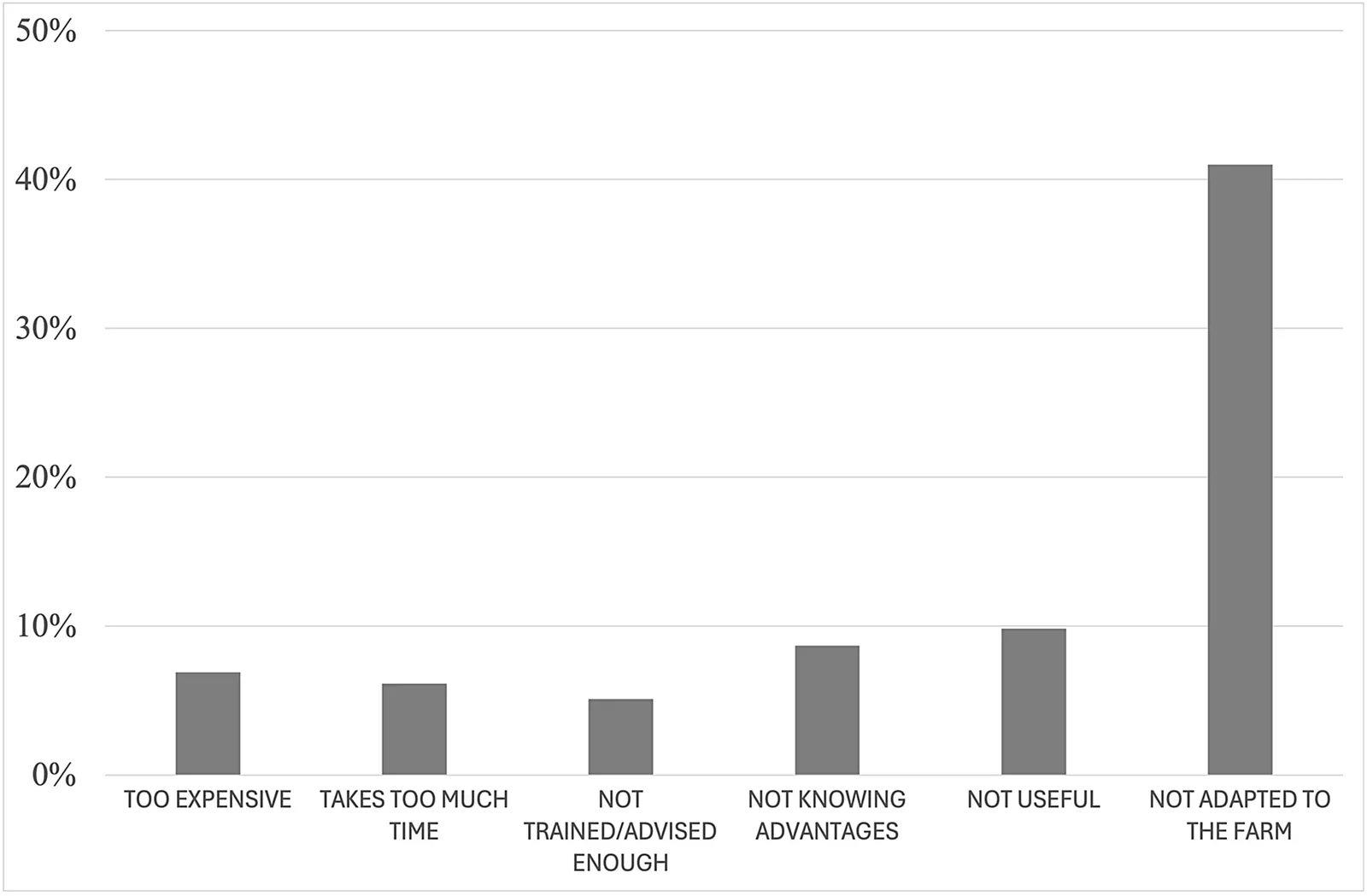
**Frequency of reasons for the lack of implementation of biosecurity measures (percentage)**

The level of implementation among respondents of different biosecurity measures ranged between 91% for the measure ‘carcass management’ and 48% for ‘cleaning and disinfection of vehicles’ (Figure 1). The main reason for not implementing biosecurity measures was that they were considered ‘not adapted to the farm’ (Figure 2). For the measures ‘knowledge of the health status of incoming animals or application of quarantine’ and ‘end-line drinking water analysis’, the majority of respondents either selected ‘other reasons’ not listed in the survey or indicated uncertainty about their reasons for non-implementation. For the measures ‘visitor access to animals is restricted and registered’, ‘usage of protective clothes/boot dips/baths at entry to animal areas’, ‘disinfect barn structures between batches of pigs/poultry using same accommodation’ and ‘prevent contact between pigs/poultry and other animals on the premises’, more than 50% of respondents considered these measures not adapted to their farms. There was a significant difference between vets/advisors and farmers in the perception of implementation for several measures. Farmers felt that the level of implementation of ‘health status of incoming animals is known or quarantine is applied’ and ‘feed storage protection to prevent contamination’ was greater than that of vets/advisors. On the other hand, vets/advisors perceived a greater level of implementation for measures such as ‘protective clothes/boot dips/baths at entry to animal areas’, ‘prevent contact between pigs/poultry and other animals on the premises’ and ‘vaccination’ than did farmers (Appendix B.2).

**Figure 3:**
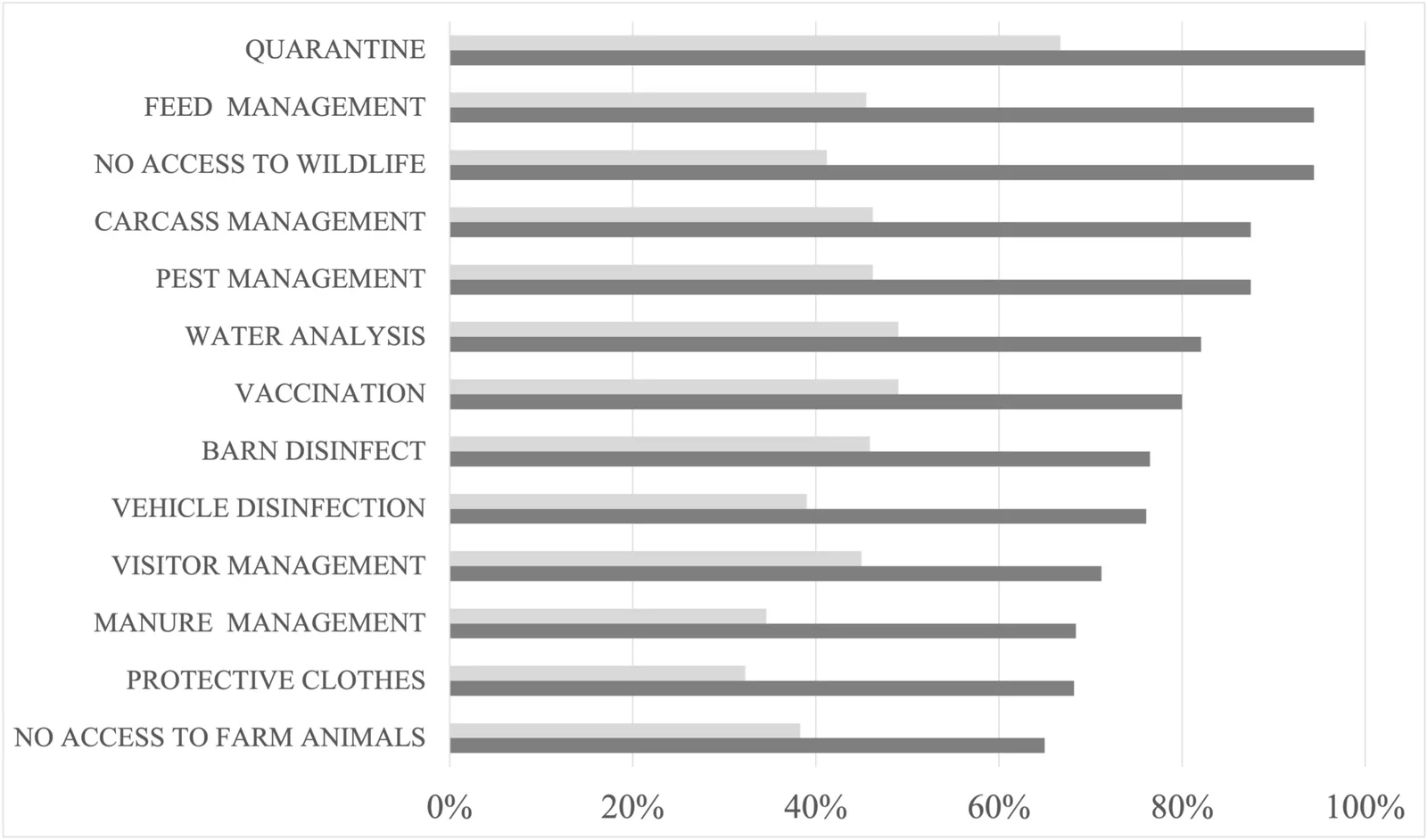
**Perception of the effectiveness of selected biosecurity measures among participants not implementing them (percentage; dark grey represents vets and advisors, and light grey represents farmers)**

When asked whether non-implemented measures would be effective for mitigating disease risk, knowledge of the ‘health status of incoming animals/quarantine’ was considered the most effective measure (83% of respondents agreed/strongly agreed with the statement), whereas using protective clothes/boot dips/baths when entering animal areas was deemed the least effective (40% of respondents agreed/strongly agreed with the statement; Figure 3). Biosecurity measures were generally perceived as more effective by vets and advisors than by farmers, except for the measure ‘carcass removal and disposal’, where there were not enough cases to run the analysis (Appendix B.3).

### Concerns regarding disease control measures and their potential consequences

**Figure 4:**
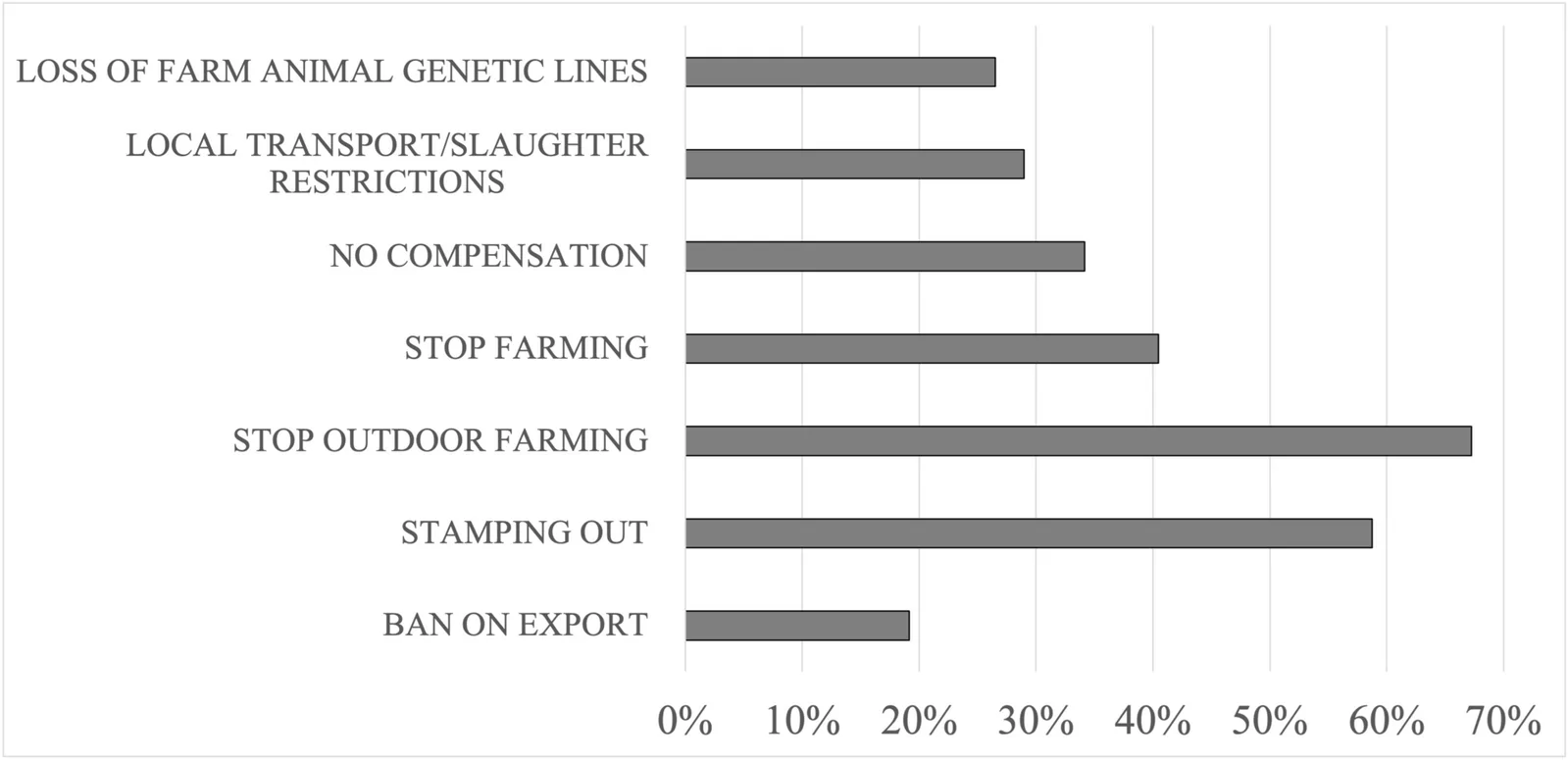
**Concerns for potential consequences of disease control measures.** The average value for pig and poultry farmers (percentage; multiple answers were possible).

The control measure perceived as most concerning by the respondents was the ban on outdoor farming (68.5% for poultry, 66% for pigs), followed by compulsory culling in the event of disease outbreaks (‘stamping out measures’: 59.9% for pigs, 57.6% for poultry). Movement restrictions, lack of compensation (or compensation based on conventional farming), farm abandonment and loss of valuable genetic lines were identified as significant concerns by approximately 30% of participants in the event of an ASF/AI outbreak (Figure 4). The least concerning control measure was a ban on exports (23.1% and 15.2% for pigs and poultry, respectively). Statistical comparisons between pig and poultry farmers were not prioritized, as 28.8% of respondents were involved in both types of farming, limiting the ability to clearly separate the two groups without introducing bias.

### Animal welfare

**Figure 5:**
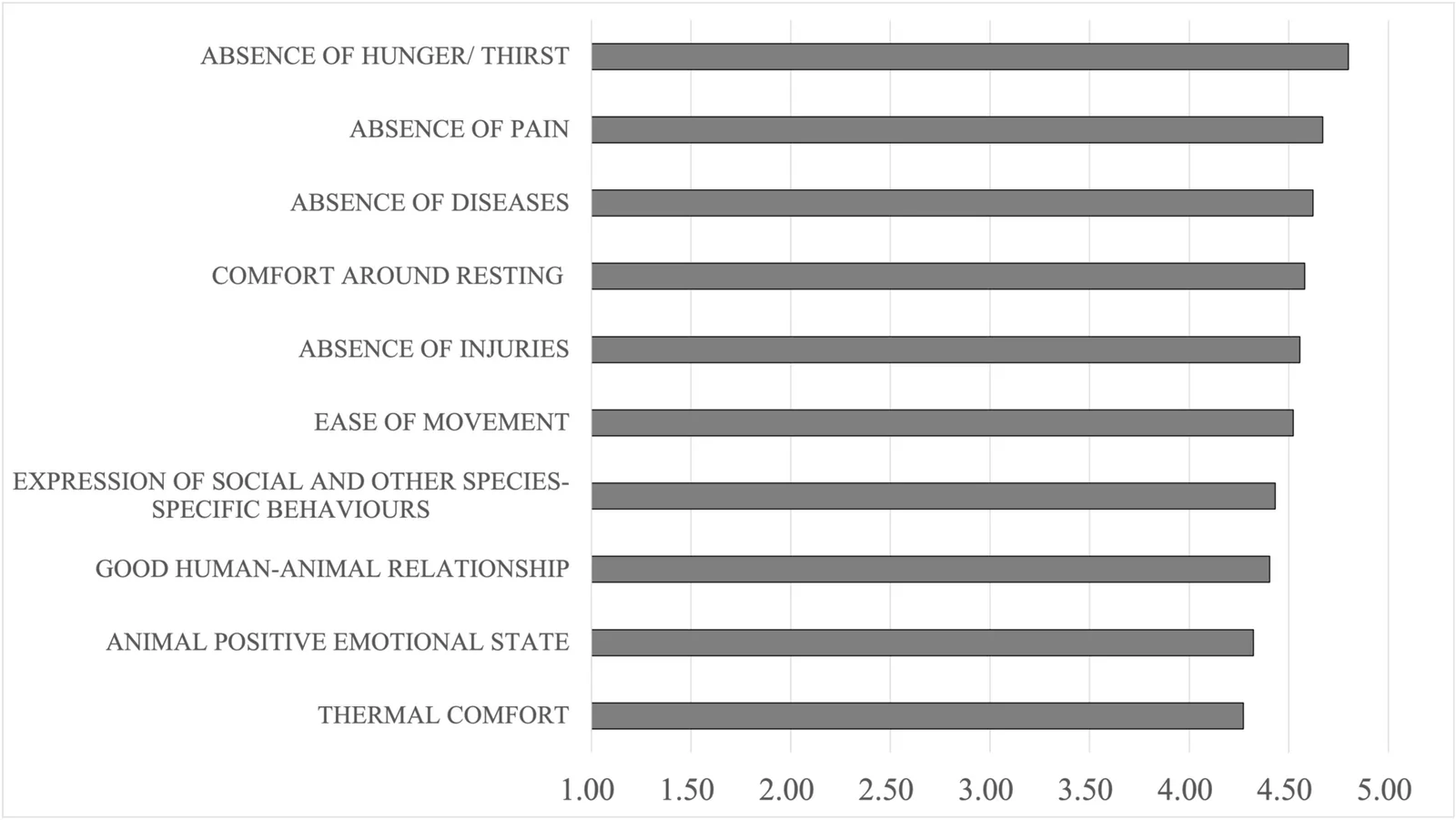
**Importance attributed to animal welfare criteria as scored by participants (average)**

The participants were asked to score animal welfare criteria, as described by Welfare Quality^®^ [41]. All criteria received consistently high importance ratings from participants, with mean scores close to the upper end of the scale (Figure 5). There was a significant difference between farmers and vets/advisors in the importance attributed to the absence of pain, freedom of movement and expression of social and other species-specific behaviours. Farmers allocated more importance to such criteria than vets/advisors did (Appendix B.4).

Considering the distinctions put forward by Lund [21] and Fraser [50], the ‘basic health and functioning’ components seemed to be ranked higher than the ‘natural living’ and ‘affective states’ items, but the rankings were not consistent. Testing this distinction did not yield clear support for the predefined three dimensions (Appendix C.1). In contrast, participants conceptualized animal welfare as a unified construct encompassing elements of health, natural living and affective states (Appendix C.2). With respect to alternative latent dimensions, it was possible to observe the presence of two latent dimensions, albeit with an eigenvalue threshold of 0.9. In this latter case, the first dimension was centred on health-related issues, including the following variables: absence of hunger/thirst, comfort around rest, thermal comfort, absence of injuries, absence of diseases and absence of pain. The second dimension focussed on natural behaviours, comprising ease of movement, good human–animal relationships and the expression of social and other species-specific behaviours. Analysing the two summative (additive) indices, farmers scored significantly higher than veterinarians/advisors did on the latter (in terms of natural and behavioural aspects), indicating that they place greater emphasis on these aspects of animal welfare. (Appendix C.3).

### Impact of biosecurity measures on animal health and welfare

Biosecurity measures, as currently required by competent authorities, were deemed to have a limited impact on present and future animal health (AH) and animal welfare (AW) according to the respondents (Table 5). In fact, more than one third of the respondents disagreed/strongly disagreed that the biosecurity measures currently required by competent authorities are leading, or will lead, to better AH and AW both in the long and short term. The effect of the implementation of biosecurity measures was considered more detrimental to animal welfare than to animal health in organic/extensive/small-scale farming systems across all the time frames considered. However, the impact on both animal health and welfare was perceived to improve when respondents considered longer time horizons (e.g. comparing ‘has already led to better AH/AW’ with ‘will lead to better AH/AW in the short term’ and ‘will lead to better AH/AW in the long term’).

**Table 5:** **Perception of the positive impact of biosecurity measures on animal health (AH) and animal welfare (AW) in small-scale, organic, extensive systems (%) at different time steps: long term (LT), short term (ST), up to now (ALREADY) and with no specific time reference (LEADS)**

	LT AH	ST AH	ALREADY AH	LEADS AH	LT AW	ST AW	ALREADY AW	LEADS AW
Disagree	18.4	21.1	25.4	20.2	25.4	27.6	27.6	25
2	19.7	18	15.8	17.1	18.9	14.9	18	16.2
3	23.2	26.3	23.2	21.9	21.5	28.9	19.3	21.5
4	26.8	25	21.9	29.4	23.2	19.3	21.5	24.1
Agree	11.8	9.6	13.6	11.4	11	9.2	13.6	13.2
Total	100	100	100	100	100	100	100	100

In addition, while both groups shared perceptions of the effects of biosecurity measures on animal health and welfare, farmers held significantly stronger negative perceptions than veterinarians and advisors did regarding the impact of these measures, as currently mandated by competent authorities (Appendix D).

## Discussion

This study investigates, through a survey, how farmers, veterinarians and advisors across six European countries view standard biosecurity measures, their effectiveness and their relationship with animal welfare in alternative farms. Under the current epidemiological circumstances related to the spread and intensity of ASF and AI outbreaks, biosecurity is considered the ‘first line of defence’ [51] for safeguarding health in flocks and herds on farms. Its effectiveness also determines the capacity to reduce cross-species transmission risks and to safeguard public health. A growing body of literature portrays alternative farmers as less compliant with standard biosecurity protocols and therefore at greater theoretical risk for disease introduction and spread [24, 25, 31, 32, 52].Yet, there is limited empirical evidence to suggest that these systems experience a higher actual incidence of disease than conventional systems do [21]. Biosecurity protocols were initially developed for conventional pig and poultry farms to prevent and control the introduction and spread of disease in facilities where animals are stocked at high densities and to mitigate the devastating economic consequences for the industry sector as a whole [26]. However, when alternative systems are managed according to organic or low-input principles, the implementation of biosecurity measures poses distinctive challenges compared with conventional intensive systems [28]. A purely health-driven approach to biosecurity risks overlooking the complex interactions between animal health, animal welfare, environmental management and farming livelihoods that characterize alternative livestock systems. In our study, even though respondents demonstrated a strong sense of responsibility and knowledge about biosecurity, similar to what has been reported in previous studies in different farming systems [47, 48], practical application was reported as limited (e.g. ‘measure not adapted to the farm’; Figure 1A) owing to incompatibility with the specific contexts and core values of these farming systems. In other words, farmers, veterinarians and advisors in our sample seemed to not be in the position nor willing to fully support the implementation of biosecurity measures and consequently the overall animal health policy goal. From a policy-oriented perspective, as shown in different settings [53], this finding is relevant because policy implementation relies on the response of properly and timely engaged target groups, whose actions or behavioural changes are key to the achievement of shared policy goals.

Although educational efforts may have successfully transferred a sense of responsibility and general awareness among respondents about animal health and disease prevention measures, this alone was probably insufficient, even among veterinarians and advisors working in alternative systems, for ensuring effective biosecurity implementation due to practical and context-based reasons, similar to what has been reported by several studies carried out in ‘marginal’ areas [36, 37, 54, 55], underscoring that biosecurity measures designed for intensive operations frequently fail to consider the spatial and operational characteristics of alternative farming systems. For example, core biosecurity measures, seldomly reported to be implemented (Figure 1A), as well as perceived to be ineffective (Figure 2), were related to cleaning and disinfection. While protective clothes could be easily used on farms, physical barriers, baths, showers and vehicle disinfection platforms in extensive settings may have been perceived as impractical, ineffective or even contrary to environmental/landscape protection regulations. Interestingly, such measures have been reported to be rarely implemented even in more intensive poultry settings [46]. Similarly, given the mixed-species nature of many small-scale farms, measures such as species segregation and controlled contact may be not desirable, as such systems seek ecological balance and resource efficiency. In addition, the closed-cycle farming practices typical of these systems could mitigate risks related to multi-species farming by limiting the movements of animals and vehicles entering the farm and therefore the introduction of pathogens (and the need to build vehicle cleaning and disinfection stations at the farm entrance).

The sense of responsibility for biosecurity was also mirrored by the high ranking of ‘health and biological functioning’ components resulting from the welfare criteria scoring question of the questionnaire. While freedom from disease and injury, plus food, water, shelter and other necessities of life, are well-known concerns for veterinarians and animal producers in the discourse on animal welfare [56, 57], in our study, farmers placed greater emphasis on the natural living and emotional states of animals, prioritizing welfare components that are more similar to those of consumers/citizens [58, 59]. Beyond individual welfare criteria, farmers and veterinarians appeared to differ in their overall welfare framework. While both groups considered health important, farmers placed significantly greater emphasis on behavioural freedom, species-specific behaviours and natural living conditions, suggesting a broader conception of animal welfare that extends beyond health and biological functioning alone. Similar results were also reported by Lund [60] when interviewing Swedish ‘pioneers of organic farming’. In our study, small-scale, organic and extensive farmers believed that animal welfare was certainly connected to health but could not be limited to it, expanding the concept to behavioural and emotional aspects. This point may represent the ultimate motivation for alternative farming as well as a resilience strategy in the face of environmental and sanitary disturbances [42]. The potential inability to adhere to the core principles of alternative farming (i.e. outdoor access) was in fact the greatest concern of the respondents in the event of an outbreak and possibly the reason for the lack of trust in biosecurity policies and their positive impact on animal health and welfare. The respondents expressed significant apprehension about restrictive disease control measures, such as bans on outdoor farming or compulsory culling during outbreaks. Over two thirds of the farmers were concerned that these measures, often seen as misaligned with the principles of alternative farming, could undermine their ability to uphold free-range, mixed-farming practices, allowing the expression of the species’ behaviour outdoors. However, similar to what has been reported by Klein et al. [48] for outdoor pig farmers, there was limited concern regarding possible bans on export, highlighting the different distribution and sales channels and thus the different role alternative farmers, compared with more intensive ones, may play in local food systems [23]. In this sense, it was also interesting to see how cost was not reported to be a relevant reason for not implementing biosecurity measures on farms (Figure 1B), supporting the hypothesis that measures were not implemented, because they were perceived, especially by farmers, as top-down impositions with negative impacts on animal health and welfare (Table 5).

These findings suggest that biosecurity is not perceived only as a technical protocol but also in relation to farming values, practical constraints and animal welfare priorities. As argued by Enticott et al. [61] and Hinchliffe et al. [29, 30], disease control is shaped not only by pathogen transmission but also by the social, economic and cultural contexts in which livestock farming take place. In this framework, veterinary science remains a crucial basis for risk assessment, but its application in alternative farming systems may require greater attention to the ecological and practical logic of the farm. For example, practices such as mixed-species farming or outdoor access should not necessarily be interpreted as a rejection of biosecurity but rather as elements that farmers evaluate in relation to broader objectives, including animal welfare, resource efficiency and farm resilience. Our results suggest that animal welfare may represent an important lens through which farmers evaluate the acceptability and appropriateness of biosecurity measures in alternative livestock systems. We argue that the farmers’ emphasis on natural living and emotional states is what allows them to bridge the gap between sanitary requirements and the reality of alternative farming. Expanding the biosecurity discourse from a strictly health-driven focus towards one that also considers One Welfare [62] perspectives may help to better account for the links between animal health, behavioural opportunities, emotional states and farm-level feasibility.

The understanding of animal welfare centred on health and biological functioning by veterinarians and agricultural professionals [60, 63] may, in fact, have led to the design and implementation of biosecurity measures and policies that fail to recognize the contribution of behavioural and emotional components in ensuring better animal health. This disparity may contribute to the limited resonance of biosecurity measures with farmers in small-scale, extensive and organic systems, as these measures often fail to address the broader welfare components these farmers prioritize. These findings support the value of complementing One Health approaches with One Welfare perspectives when designing animal health policies, which is critical for fostering environments that promote positive animal experiences and that recognize the contribution of behavioural and emotional components to healthy lives [42]. The observed divergence between farmers and veterinarians is particularly relevant in this respect, as it suggests that different stakeholder groups may prioritize different welfare dimensions when evaluating the appropriateness of biosecurity measures.

While the recent EU framework regulation on animal health highlighted the positive relationship between health and welfare and the need to foster inclusive decision-making with stakeholders [38], it has been argued that to reach more coherence across policy domains (e.g. health and welfare), the participation of alternative farmers should be reinforced [23]. By directly involving stakeholders – those who are the most familiar with the daily routine of alternative farming – in the policy-making process, it may be possible to develop biosecurity measures that are not only scientifically robust but also practically applicable and widely accepted .

As seen in the management of sheep scab in Scotland [37] and ASF in Corsica [36], our findings are consistent with previous studies suggesting that place-based approaches may facilitate the implementation of biosecurity measures in non-intensive systems. By moving the scale of policy design from the national to the territorial level, authorities can involve stakeholders in defining measures that are both epidemiologically sound and practically feasible for outdoor and organic systems. In fact, the engagement of small-scale, extensive and organic farmers in the animal farming policy-making process would allow the integration of values, such as the importance of natural living and positive emotional states, which are widely shared by consumers but are currently under-represented in animal health and welfare policies. This integration may serve as a stepping stone towards achieving better policy coherence and ensuring effective implementation across the diverse farming systems that characterize the European agricultural environment.

While this study provides a novel overview of biosecurity and welfare views in alternative farming systems, several limitations must be acknowledged. First, we acknowledge the methodological implications of using Likert-scale statements. While standard in knowledge, attitudes, and practices (KAP) studies, it presents inherent limitations when investigating non-mainstream populations. Standardized surveys may fail to capture how actors truly conceptualize their reality if the questionnaire’s language is rooted in a dominant discourse [64]. In our case, the survey was strongly influenced by mainstream narrative based on the intensive-farming language. This approach inherently limits the depth of individual expression compared with qualitative interviews, whereas we have integrated the interpretation of open-ended questions included in the survey. Nonetheless, we call for a more qualitative approach for a better understanding of alternative farming practices, when it comes to biosecurity. While open-ended responses provided useful contextual insights, they cannot substitute for in-depth qualitative inquiry. Furthermore, the present study captures perceptions and self-reported practices rather than objective measures of disease occurrence, biosecurity effectiveness or animal welfare outcomes. Future research combining survey methods with qualitative approaches and field-based assessments would therefore provide a more comprehensive understanding of biosecurity in alternative livestock systems.

Second, although the study involved participants from multiple European countries to capture a diverse range of national contexts, the analysis does not disaggregate results by country or by type of farming. This is primarily due to sample limitations; the sample size does not yield sufficient statistical power within national cohorts to support robust comparative analysis. While the observed uniformity across countries might suggest a certain homogeneity in how alternative farmers and veterinarians perceive biosecurity, this should be interpreted with caution. In addition, the study dealt with two different livestock species which share an increasing farming intensification but certainly have their own peculiarities. It is however possible that the shared challenges of alternative farming – such as outdoor access and lower-input systems – transcend national and species-specific differences, creating a common ‘alternative farming identity’ across borders. Future research with larger, balanced species- specific and national samples is needed to explore whether specific livestock species, local regulations and cultural nuances create significant variations that this study could not statistically detect.

## Conclusions

This study highlights that while farmers, veterinarians and advisors in organic, extensive and small-scale systems demonstrate a strong sense of responsibility for biosecurity, the practical implementation of measures remains challenging owing to the perceived incompatibility of standardized practices with the realities and values of alternative farming systems.

A key finding of this study is that farmers consistently placed greater emphasis than veterinarians and advisors on behavioural freedom, natural living and species-specific behaviours, highlighting different conceptions of animal welfare among stakeholder groups involved in alternative livestock systems. Furthermore, the high level of concern identified regarding ‘stamping out’ and the potential prohibition of outdoor farming suggests that current top-down policies do not sufficiently account for the systemic vulnerabilities or the intrinsic motivations of alternative farmers. A participatory approach that leverages diverse stakeholders’ perspectives could lead to policies that are both scientifically robust and practically applicable, ensuring greater acceptance and effectiveness in achieving biosecurity goals, bridging the gap between regulatory requirements and on-farm realities.

To increase biosecurity compliance and efficacy, future research should explore whether co-designed approaches involving farmers, veterinarians and advisors could improve the acceptability and effectiveness of biosecurity measures by expanding the discourse from a strict health-driven focus towards one that better accounts for the links between animal health, animal welfare and the overall socio-economic, environmental and cultural farming context.

## Supplementary information

**Additional file 1**. **Appendix**.

## Data Availability

‘Stakeholder Survey on Biosecurity and Animal Welfare in EU Organic, Extensive, and Small-Scale Pig and Poultry Farming Systems’ data were deposited into the Research Data Unipd under accession number 1924 and are available at the following link: https://doi.org/10.25430/RESEARCHDATA.CAB.UNIPD.IT.00001924.
