# Supplementary material for: Life worth living or healthy lives? Stakeholder views on biosecurity and animal welfare in EU organic, extensive and small-scale pig and poultry farming systems
Source: Vet Res. 2026 Nov 10;57:200. doi: 10.1186/s13567-026-01865-0 (PMC13753137; doi:10.1186/s13567-026-01865-0)
Supplement: Supplementary file 1 [file 13567_2026_1865_MOESM1_ESM.docx]

**Appendix A - Excerpt of the Survey**

**Animal health and biosecurity measures**

1. How often do you implement / see implemented the following measures? (1 never - 5 always)
   - Health status of incoming animals is known or quarantine is applied
   - Visitor access to animals is restricted and registered
   - Cleaning and disinfection of vehicles before entering the premises
   - Protective clothes/ Boot dips/baths at entry to animal areas
   - Disinfect barn structures between batches of pigs/poultry using same accommodation
   - Feed storage protection to prevent contamination
   - End-line drinking water analysis
   - Control rodents
   - Take measures to stop wildlife accessing animals or animal pens
   - Prevent contact between pigs/poultry and other animals on the premises
   - Carcass removal and disposal
   - Manure is managed (stored and removed) avoiding contamination of animal housing areas
   - Vaccination regime
2. Could you explain the reason?

- too expensive,
- takes too much time,
- not trained/advised enough,
- not knowing advantages,
- no useful,
- not adapted to the farm,
- other reason to be specified,
- unknown

3. Do you think the measure would be an efficient biosecurity measure in your farm/the farms where you work? 1 strongly disagree – 5 strongly agree

**Appendix B *– Associations between different biosecurity metrics and job condition.***

*Appendix B.1 – Associations between biosecurity statements and professions*

**Table B1.** Familiarity with biosecurity by profession. *

|  |  | **Other** | **Farmer** | **Total** |
| --- | --- | --- | --- | --- |
| Disagree | Count | 11 | 28 | 39 |
|  | Expected Count | 16.2 | 22.8 | 39.0 |
| Agree | Count | 85 | 107 | 192 |
|  | Expected Count | 79.8 | 112.2 | 192.0 |
| Total | Count | 96 | 135 | 231 |
|  | Expected Count | 96.0 | 135.0 | 231.0 |

** X^2^(1) = 3.445, P = .063*

**Table B2. “**I feel responsible for biosecurity” by profession. *

|  |  | **Other** | **Farmer** | **Total** |
| --- | --- | --- | --- | --- |
| Disagree | Count | 14 | 21 | 35 |
|  | Expected Count | 14.5 | 20.5 | 35.0 |
| Agree | Count | 82 | 114 | 196 |
|  | Expected Count | 81.5 | 114.5 | 196.0 |
| Total | Count | 96 | 135 | 231 |
|  | Expected Count | 96.0 | 135.0 | 231.0 |

** X^2^(1) = 0.041, P = .839*

**Table B3.** “I have enough information to implement biosecurity” by profession. *

|  |  | **Other** | **Farmer** | **Total** |
| --- | --- | --- | --- | --- |
| Disagree | Count | 21 | 44 | 65 |
|  | Expected Count | 27.0 | 38.0 | 65.0 |
| Agree | Count | 75 | 91 | 166 |
|  | Expected Count | 69.0 | 97.0 | 166.0 |
| Total | Count | 96 | 135 | 231 |
|  | Expected Count | 96.0 | 135.0 | 231.0 |

** X^2^(1) = 3.187, P = .074*

**Table B4.** “I think biosecurity can be improved in my company” by profession. *

|  |  | **Other** | **Farmer** | **Total** |
| --- | --- | --- | --- | --- |
| Disagree | Count | 28 | 75 | 103 |
|  | Expected Count | 42.8 | 60.2 | 103.0 |
| Agree | Count | 68 | 60 | 128 |
|  | Expected Count | 53.2 | 74.8 | 128.0 |
| Total | Count | 96 | 135 | 231 |
|  | Expected Count | 96.0 | 135.0 | 231.0 |

** X^2^(1) = 15.813, P < .001****

*Appendix B.2 – Associations between biosecurity measures and job conditions*

**Table B9. “**Quarantine” by job condition. *

|  |  | **Other** | **Farmer** | **Total** |
| --- | --- | --- | --- | --- |
| Rarely | Count | 20 | 15 | 35 |
|  | Expected Count | 14.5 | 20.5 | 35.0 |
| Often | Count | 76 | 120 | 196 |
|  | Expected Count | 81.5 | 114.5 | 196.0 |
| Total | Count | 96 | 135 | 231 |
|  | Expected Count | 96.0 | 135.0 | 231.0 |

** X^2^(1) = 4.125, P = .042**

**Table B10. “**Visitor Management” by job condition. *

|  |  | **Other** | **Farmer** | **Total** |
| --- | --- | --- | --- | --- |
| Rarely | Count | 24 | 40 | 64 |
|  | Expected Count | 26.6 | 37.4 | 64.0 |
| Often | Count | 72 | 95 | 167 |
|  | Expected Count | 69.4 | 97.6 | 167.0 |
| Total | Count | 96 | 135 | 231 |
|  | Expected Count | 96.0 | 135.0 | 231.0 |

** X^2^(1) = .600, P = .438*

**Table B11. “**Vehicle disinfection” by job condition. *

|  |  | **Other** | **Farmer** | **Total** |
| --- | --- | --- | --- | --- |
| Rarely | Count | 46 | 77 | 123 |
|  | Expected Count | 51.1 | 71.9 | 123.0 |
| Often | Count | 50 | 58 | 108 |
|  | Expected Count | 44.9 | 63.1 | 108.0 |
| Total | Count | 96 | 135 | 231 |
|  | Expected Count | 96.0 | 135.0 | 231.0 |

** X^2^(1) = 1.875, P = .171*

**Table B12. “**Protective clothes” by job condition. *

|  |  | **Other** | **Farmer** | **Total** |
| --- | --- | --- | --- | --- |
| Rarely | Count | 22 | 65 | 87 |
|  | Expected Count | 36.2 | 50.8 | 87.0 |
| Often | Count | 74 | 70 | 144 |
|  | Expected Count | 59.8 | 84.2 | 144.0 |
| Total | Count | 96 | 135 | 231 |
|  | Expected Count | 96.0 | 135.0 | 231.0 |

** X^2^(1) = 15.213, P <.001****

**Table B13. “**Barn Disinfect” by job condition. *

|  |  | **Other** | **Farmer** | **Total** |
| --- | --- | --- | --- | --- |
| Rarely | Count | 17 | 37 | 54 |
|  | Expected Count | 22.4 | 31.6 | 54.0 |
| Often | Count | 79 | 98 | 177 |
|  | Expected Count | 73.6 | 103.4 | 177.0 |
| Total | Count | 96 | 135 | 231 |
|  | Expected Count | 96.0 | 135.0 | 231.0 |

** X^2^(1) = 2.947, P = .086*

**Table B14. “**Feed Management” by job condition. *

|  |  | **Other** | **Farmer** | **Total** |
| --- | --- | --- | --- | --- |
| Rarely | Count | 18 | 11 | 29 |
|  | Expected Count | 12.1 | 16.9 | 29.0 |
| Often | Count | 78 | 124 | 202 |
|  | Expected Count | 83.9 | 118.1 | 202.0 |
| Total | Count | 96 | 135 | 231 |
|  | Expected Count | 96.0 | 135.0 | 231.0 |

** X^2^(1) = 5.744, P = .017**

**Table B15. “**Water analysis” by job condition. *

|  |  | **Other** | **Farmer** | **Total** |
| --- | --- | --- | --- | --- |
| Rarely | Count | 29 | 53 | 82 |
|  | Expected Count | 34.1 | 47.9 | 82.0 |
| Often | Count | 67 | 82 | 149 |
|  | Expected Count | 61.9 | 87.1 | 149.0 |
| Total | Count | 96 | 135 | 231 |
|  | Expected Count | 96.0 | 135.0 | 231.0 |

** X^2^(1) = 2.007, P = .157*

**Table B16. “**Pest Management” by job condition. *

|  |  | **Other** | **Farmer** | **Total** |
| --- | --- | --- | --- | --- |
| Rarely | Count | 16 | 13 | 29 |
|  | Expected Count | 12.1 | 16.9 | 29.0 |
| Often | Count | 80 | 122 | 202 |
|  | Expected Count | 83.9 | 118.1 | 202.0 |
| Total | Count | 96 | 135 | 231 |
|  | Expected Count | 96.0 | 135.0 | 231.0 |

** X^2^(1) = 2.531, P = .112*

**Table B17. “**No Access to Wildlife” by job condition. *

|  |  | **Other** | **Farmer** | **Total** |
| --- | --- | --- | --- | --- |
| Rarely | Count | 18 | 34 | 52 |
|  | Expected Count | 21.6 | 30.4 | 52.0 |
| Often | Count | 78 | 101 | 179 |
|  | Expected Count | 74.4 | 104.6 | 179.0 |
| Total | Count | 96 | 135 | 231 |
|  | Expected Count | 96.0 | 135.0 | 231.0 |

** X^2^(1) = 1.332, P = .248*

**Table B18. “**No Access to Farm Animals” by job condition. *

|  |  | **Other** | **Farmer** | **Total** |
| --- | --- | --- | --- | --- |
| Rarely | Count | 20 | 47 | 67 |
|  | Expected Count | 27.8 | 39.2 | 67.0 |
| Often | Count | 76 | 88 | 164 |
|  | Expected Count | 68.2 | 95.8 | 164.0 |
| Total | Count | 96 | 135 | 231 |
|  | Expected Count | 96.0 | 135.0 | 231.0 |

** X^2^(1) = 5.326, P = .021**

**Table B19. “**Carcass Management” by job condition. *

|  |  | **Other** | **Farmer** | **Total** |
| --- | --- | --- | --- | --- |
| Rarely | Count | 8 | 13 | 21 |
|  | Expected Count | 8.7 | 12.3 | 21.0 |
| Often | Count | 88 | 122 | 210 |
|  | Expected Count | 87.3 | 122.7 | 210.0 |
| Total | Count | 96 | 135 | 231 |
|  | Expected Count | 96.0 | 135.0 | 231.0 |

** X^2^(1) = .114, P = .736*

**Table B20. “**Manure Management” by job condition. *

|  |  | **Other** | **Farmer** | **Total** |
| --- | --- | --- | --- | --- |
| Rarely | Count | 19 | 26 | 45 |
|  | Expected Count | 18.7 | 26.3 | 45.0 |
| Often | Count | 77 | 109 | 186 |
|  | Expected Count | 77.3 | 108.7 | 186.0 |
| Total | Count | 96 | 135 | 231 |
|  | Expected Count | 96.0 | 135.0 | 231.0 |

** X^2^(1) = .010, P = .920*

**Table B21. “**Vaccination” by job condition. *

|  |  | **Other** | **Farmer** | **Total** |
| --- | --- | --- | --- | --- |
| Rarely | Count | 19 | 26 | 45 |
|  | Expected Count | 18.7 | 26.3 | 45.0 |
| Often | Count | 77 | 109 | 186 |
|  | Expected Count | 77.3 | 108.7 | 186.0 |
| Total | Count | 96 | 135 | 231 |
|  | Expected Count | 96.0 | 135.0 | 231.0 |

** X^2^(1) = 7.566, P = .006***

*Appendix B.3– Associations between perceived effectiveness of measures and job condition*

**Table B22. “**Quarantine” by job condition. *

|  |  | **Other** | **Farmer** | **Total** |
| --- | --- | --- | --- | --- |
| Non efficient | Count | 0 | 5 | 5 |
|  | Expected Count | 2.9 | 2.1 | 5.0 |
| Partially/or efficient | Count | 20 | 10 | 30 |
|  | Expected Count | 17.1 | 12.9 | 30.0 |
| Total | Count | 20 | 15 | 35 |
|  | Expected Count | 20.0 | 15.0 | 35.0 |

** X^2^(1) = 7.778, P = .005***

**Table B23. “**Visitor Management” by job condition. *

|  |  | **Other** | **Farmer** | **Total** |
| --- | --- | --- | --- | --- |
| Non efficient | Count | 5 | 22 | 27 |
|  | Expected Count | 10.1 | 16.9 | 27.0 |
| Partially/or efficient | Count | 19 | 18 | 37 |
|  | Expected Count | 13.9 | 23.1 | 37.0 |
| Total | Count | 24 | 40 | 64 |
|  | Expected Count | 24.0 | 40.0 | 64.0 |

** X^2^(1) = 7.179, P = .007***

**Table B24. “**Vehicle disinfection” by job condition. *

|  |  | **Other** | **Farmer** | **Total** |
| --- | --- | --- | --- | --- |
| Non efficient | Count | 11 | 47 | 58 |
|  | Expected Count | 21.7 | 36.3 | 58.0 |
| Partially/or efficient | Count | 35 | 30 | 65 |
|  | Expected Count | 24.3 | 40.7 | 65.0 |
| Total | Count | 46 | 77 | 123 |
|  | Expected Count | 46.0 | 77.0 | 123.0 |

** X^2^(1) = 15.928, P < .001****

**Table B25. “**Protective clothes” by job condition. *

|  |  | **Other** | **Farmer** | **Total** |
| --- | --- | --- | --- | --- |
| Non efficient | Count | 7 | 44 | 51 |
|  | Expected Count | 12.9 | 38.1 | 51.0 |
| Partially/or efficient | Count | 15 | 21 | 36 |
|  | Expected Count | 9.1 | 26.9 | 36.0 |
| Total | Count | 22 | 65 | 87 |
|  | Expected Count | 22.0 | 65.0 | 87.0 |

** X^2^(1) = 8.721, P = .003***

**Table B26. “**Barn Disinfect” by job condition. *

|  |  | **Other** | **Farmer** | **Total** |
| --- | --- | --- | --- | --- |
| Non efficient | Count | 4 | 20 | 24 |
|  | Expected Count | 7.6 | 16.4 | 24.0 |
| Partially/or efficient | Count | 13 | 17 | 30 |
|  | Expected Count | 9.4 | 20.6 | 30.0 |
| Total | Count | 17 | 37 | 54 |
|  | Expected Count | 17.0 | 37.0 | 54.0 |

** X^2^(1) = 4.396, P = .036**

**Table B27. “**Feed Management” by job condition. *

|  |  | **Other** | **Farmer** | **Total** |
| --- | --- | --- | --- | --- |
| Non efficient | Count | 1 | 6 | 7 |
|  | Expected Count | 4.3 | 2.7 | 7.0 |
| Partially/or efficient | Count | 17 | 5 | 22 |
|  | Expected Count | 13.7 | 8.3 | 22.0 |
| Total | Count | 18 | 11 | 29 |
|  | Expected Count | 18.0 | 11.0 | 29.0 |

** X^2^(1) = 8.949, P = .003***

**Table B28. “**Water analysis” by job condition. *

|  |  | **Other** | **Farmer** | **Total** |
| --- | --- | --- | --- | --- |
| Non efficient | Count | 5 | 26 | 31 |
|  | Expected Count | 11.0 | 20.0 | 31.0 |
| Partially/or efficient | Count | 23 | 25 | 48 |
|  | Expected Count | 17.0 | 31.0 | 48.0 |
| Total | Count | 28 | 51 | 79 |
|  | Expected Count | 28.0 | 51.0 | 79.0 |

** X^2^(1) = 8.318, P = .004***

**Table B29. “**Pest Management” by job condition. *

|  |  | **Other** | **Farmer** | **Total** |
| --- | --- | --- | --- | --- |
| Non efficient | Count | 2 | 7 | 9 |
|  | Expected Count | 5.0 | 4.0 | 9.0 |
| Partially/or efficient | Count | 14 | 6 | 20 |
|  | Expected Count | 11.0 | 9.0 | 20.0 |
| Total | Count | 16 | 13 | 29 |
|  | Expected Count | 16.0 | 13.0 | 29.0 |

** X^2^(1) = 5.729, P = .017**

**Table B30. “**No Access to Wildlife” by job condition. *

|  |  | **Other** | **Farmer** | **Total** |
| --- | --- | --- | --- | --- |
| Non efficient | Count | 1 | 20 | 21 |
|  | Expected Count | 7.3 | 13.7 | 21.0 |
| Partially/or efficient | Count | 17 | 14 | 31 |
|  | Expected Count | 10.7 | 20.3 | 31.0 |
| Total | Count | 18 | 34 | 52 |
|  | Expected Count | 18.0 | 34.0 | 52.0 |

** X^2^(1) = 13.871, P < .001****

**Table B31. “**No Access to Farm Animals” by job condition. *

|  |  | **Other** | **Farmer** | **Total** |
| --- | --- | --- | --- | --- |
| Non efficient | Count | 7 | 29 | 36 |
|  | Expected Count | 10.7 | 25.3 | 36.0 |
| Partially/or efficient | Count | 13 | 18 | 31 |
|  | Expected Count | 9.3 | 21.7 | 31.0 |
| Total | Count | 20 | 47 | 67 |
|  | Expected Count | 20.0 | 47.0 | 67.0 |

** X^2^(1) = 4.024, P = .045**

**Table B32. “**Carcass Management” by job condition. *

|  |  | **Other** | **Farmer** | **Total** |
| --- | --- | --- | --- | --- |
| Non efficient | Count | 1 | 7 | 8 |
|  | Expected Count | 3.0 | 5.0 | 8.0 |
| Partially/or efficient | Count | 7 | 6 | 13 |
|  | Expected Count | 5.0 | 8.0 | 13.0 |
| Total | Count | 8 | 13 | 21 |
|  | Expected Count | 8.0 | 13.0 | 21.0 |

** X^2^(1) = 3.590, P = .058*

**Table B33. “**Manure Management” by job condition. *

|  |  | **Other** | **Farmer** | **Total** |
| --- | --- | --- | --- | --- |
| Non efficient | Count | 6 | 17 | 23 |
|  | Expected Count | 9.7 | 13.3 | 23.0 |
| Partially/or efficient | Count | 13 | 9 | 22 |
|  | Expected Count | 9.3 | 12.7 | 22.0 |
| Total | Count | 19 | 26 | 45 |
|  | Expected Count | 19.0 | 26.0 | 45.0 |

** X^2^(1) = 5.021, P = .025**

**Table B34. “**Vaccination” by job condition. *

|  |  | **Other** | **Farmer** | **Total** |
| --- | --- | --- | --- | --- |
| Non efficient | Count | 4 | 26 | 30 |
|  | Expected Count | 8.5 | 21.5 | 30.0 |
| Partially/or efficient | Count | 16 | 25 | 41 |
|  | Expected Count | 11.5 | 29.5 | 41.0 |
| Total | Count | 20 | 51 | 71 |
|  | Expected Count | 20.0 | 51.0 | 71.0 |

** X^2^(1) = 5.651, P = .017**

*Appendix B.4 – Associations between animal welfare criteria and job condition*

**Table B35.** The importance of “absence of hunger/thirst” by job condition. *

| **Importance** | **Other** | **Farmer** | **Total** |
| --- | --- | --- | --- |
| 1 | 3 | 2 | 5 |
| 2 | 0 | 0 | 0 |
| 3 | 1 | 2 | 3 |
| 4 | 7 | 11 | 18 |
| 5 | 85 | 120 | 205 |
| Total | 96 | 135 | 231 |

** X2(3) = .837, P = .841*

**Table B36.** The importance of **“**Comfort around resting” by job condition. *

| **Importance** | **Other** | **Farmer** | **Total** |
| --- | --- | --- | --- |
| 1 | 3 | 2 | 5 |
| 2 | 2 | 0 | 2 |
| 3 | 2 | 4 | 6 |
| 4 | 21 | 36 | 57 |
| 5 | 68 | 93 | 161 |
| Total | 96 | 135 | 231 |

** X^2^(4) = 4.232, P = .375*

**Table B37.** The importance of **“**Ease of movement” by job condition. *

| **Importance** | **Other** | **Farmer** | **Total** |
| --- | --- | --- | --- |
| 1 | 3 | 2 | 5 |
| 2 | 4 | 0 | 4 |
| 3 | 10 | 5 | 15 |
| 4 | 27 | 24 | 51 |
| 5 | 52 | 104 | 156 |
| Total | 96 | 135 | 231 |

** X2(4) = 17.285, P = .002***

**Table B38.** The importance of “thermal comfort” by job condition. *

| **Importance** | **Other** | **Farmer** | **Total** |
| --- | --- | --- | --- |
| 1 | 2 | 1 | 3 |
| 2 | 3 | 4 | 7 |
| 3 | 9 | 21 | 30 |
| 4 | 26 | 47 | 73 |
| 5 | 56 | 62 | 118 |
| Total | 96 | 135 | 231 |

* *X2*(4) = 5.186, *P =* .269

**Table B39.** The importance of “absence of injuries” by job condition. *

| **Importance** | **Other** | **Farmer** | **Total** |
| --- | --- | --- | --- |
| 1 | 3 | 2 | 5 |
| 2 | 0 | 1 | 1 |
| 3 | 4 | 3 | 7 |
| 4 | 30 | 32 | 62 |
| 5 | 59 | 97 | 156 |
| Total | 96 | 135 | 231 |

** X^2^(4) = 4.199, P = .380*

**Table B40.** The importance of “absence of diseases” by job condition. *

| **Importance** | **Other** | **Farmer** | **Total** |
| --- | --- | --- | --- |
| 1 | 3 | 2 | 5 |
| 2 | 0 | 0 | 0 |
| 3 | 4 | 4 | 8 |
| 4 | 22 | 25 | 47 |
| 5 | 67 | 104 | 171 |
| Total | 96 | 135 | 231 |

** X^2^(3) = 1.866, P = .601*

**Table B41.** The importance of “absence of pain” by job condition. *

| **Importance** | **Other** | **Farmer** | **Total** |
| --- | --- | --- | --- |
| 1 | 2 | 2 | 4 |
| 2 | 1 | 0 | 1 |
| 3 | 7 | 1 | 8 |
| 4 | 19 | 19 | 38 |
| 5 | 67 | 113 | 180 |
| Total | 96 | 135 | 231 |

** X^2^(4) =10.984, P = .027**

**Table B42.** The importance of “good human–animal relationships” by job condition. *

| **Importance** | **Other** | **Farmer** | **Total** |
| --- | --- | --- | --- |
| 1 | 2 | 2 | 4 |
| 2 | 3 | 3 | 6 |
| 3 | 8 | 9 | 17 |
| 4 | 33 | 36 | 69 |
| 5 | 50 | 85 | 135 |
| Total | 96 | 135 | 231 |

** X^2^(4) = 2.758, P = .599*

**Table B43.** The importance of “expression of social and other species-specific behaviours” by job condition. *

| **Importance** | **Other** | **Farmer** | **Total** |
| --- | --- | --- | --- |
| 1 | 3 | 1 | 4 |
| 2 | 1 | 1 | 2 |
| 3 | 9 | 12 | 21 |
| 4 | 38 | 30 | 68 |
| 5 | 45 | 91 | 136 |
| Total | 96 | 135 | 231 |

** X^2^(4) = 11.677, P = .020**

**Table B44.** Importance of “animal positive emotional state” by job condition. *

| **Importance** | **Other** | **Farmer** | **Total** |
| --- | --- | --- | --- |
| 1 | 2 | 3 | 5 |
| 2 | 4 | 4 | 8 |
| 3 | 12 | 7 | 19 |
| 4 | 29 | 42 | 71 |
| 5 | 49 | 79 | 128 |
| Total | 96 | 135 | 231 |

** X^2^(4) = 4.470, P = .346*

**Appendix C** *– Confirmatory factor analysis on animal welfare criteria*

*Appendix C.1 – Confirmatory factor analysis using three factors*

Confirmatory factor analysis (CFA) was performed to validate the factor structure underlying the ten variables under study. We deployed a varimax rotation to increase the level of correlation with Kaiser normalization, with 3 factors explaining 76.78% of the cumulative variance.

**Table C1.** Total variance Explained*

|  | **Initial Eigenvalues** | | | **Extraction Sums of Squared Loadings** | | | **Rotation Sums of Squared Loadings** | | |
| --- | --- | --- | --- | --- | --- | --- | --- | --- | --- |
|  | **Total** | **% of Var** | **Cum %** | **Total** | **% of Var** | **Cum %** | **Total** | **% of Var** | **Cum %** |
| 1 | 6.143 | 61.432 | 61.432 | 6.143 | 61.432 | 61.432 | 3.427 | 34.267 | 34.267 |
| 2 | .969 | 9.695 | 71.127 | .969 | 9.695 | 71.127 | 2.695 | 26.946 | 61.212 |
| 3 | .566 | 5.657 | 76.784 | .566 | 5.657 | 76.784 | 1.557 | 15.572 | 76.784 |
| 4 | .558 | 5.575 | 82.360 |  |  |  |  |  |  |
| 5 | .391 | 3.908 | 86.268 |  |  |  |  |  |  |
| 6 | .346 | 3.458 | 89.726 |  |  |  |  |  |  |
| 7 | .333 | 3.326 | 93.052 |  |  |  |  |  |  |
| 8 | .266 | 2.659 | 95.711 |  |  |  |  |  |  |
| 9 | .232 | 2.318 | 98.029 |  |  |  |  |  |  |
| 10 | .197 | 1.971 | 100.000 |  |  |  |  |  |  |

** * Extraction Method: Principal component analysis*

**Table C2.** Rotated component matrix*

|  | **1** | **2** | **3** |
| --- | --- | --- | --- |
| Absence of hunger/thirst | .821 |  | .322 |
| Comfort around resting | .798 |  | .466 |
| Ease of movement | .739 | .571 |  |
| Thermal comfort | .703 |  | .795 |
| Absence of injuries | .868 | .389 | .456 |
| Absence of diseases | .821 | .323 |  |
| Absence of pain | .842 | .315 | .307 |
| Good human-animal relationship | .736 | .760 | .387 |
| Expression of social and other species-specific behaviours | .755 | .783 |  |
| Animal positive emotional state | .739 | .825 |  |

**Extraction Method: Principal component analysis. Rotation Method: Varimax with Kaiser normalization.
Rotation converged in 9 iterations. KMO=.929, Bartlett’s test of sphericity: χ²(45) = 1636.821, P < .001.*

**Table C3.** Component Transformation Matrix

| Component | **1** | **2** | **3** |
| --- | --- | --- | --- |
| 1 | .706 | .578 | .409 |
| 2 | -.444 | .811 | -.381 |
| 3 | -.552 | .087 | .829 |

*Appendix C.2 – Confirmatory factor analysis using one factor*

Factor analysis was performed to gauge the factor structure underlying the ten variables under study. When an eigenvalue above 1 is set, the latent dimension explains 61.43% of the cumulative variance.

**Table C4.** Component Matrix*

|  | **1** |
| --- | --- |
| Absence of hunger/thirst | .821 |
| Comfort around resting | .798 |
| Ease of movement | .739 |
| Thermal comfort | .703 |
| Absence of injuries | .868 |
| Absence of diseases | .821 |
| Absence of pain | .842 |
| Good human-animal relationship | .736 |
| Expression of social and other species-specific behaviours | .755 |
| Animal positive emotional state | .739 |

**Extraction Method: Principal component analysis, 1 component extracted.
 KMO=.929, Bartlett’s test of sphericity: χ²(45) = 1636.821, P < .001.*

*Appendix C.3 – Confirmatory factor analysis using two factors*

Confirmatory factor analysis (CFA) was performed to gauge the factor structure underlying the ten variables under study. We deployed both an oblimin rotation and a variamax rotation with an eigenvalue above .9, explaining 71.13% of the cumulative variance.

**Table C5.** Pattern Matrix*

|  | **1** | **2** |
| --- | --- | --- |
| Absence of hunger/thirst | .940 |  |
| Comfort around resting | .831 |  |
| Ease of movement |  | .592 |
| Thermal comfort | .824 |  |
| Absence of injuries | .747 |  |
| Absence of diseases | .745 |  |
| Absence of pain | .792 |  |
| Good human-animal relationship |  | .800 |
| Expression of social and other species-specific behaviors |  | .882 |
| Animal positive emotional state |  | .917 |

** Extraction Method: Principal component analysis. Rotation Method: Oblimin with Kaiser normalization. The rotation converges in 5 iterations. KMO=.929, Bartlett’s test of sphericity: χ²(45) = 1636,821, P < .001.*

**Table C6.** Rotated component matrix*

|  | **1** | **2** |
| --- | --- | --- |
| Absence of hunger/thirst | .846 |  |
| Comfort around resting | .777 | .313 |
| Ease of movement | .428 | .641 |
| Thermal comfort | .735 |  |
| Absence of injuries | .759 | .444 |
| Absence of diseases | .739 | .395 |
| Absence of pain | .773 | .387 |
| Good human-animal relationship | .322 | .765 |
| Expression of social and other species-specific behaviors |  | .821 |
| Animal positive emotional state |  | .836 |

** Extraction Method: Principal component analysis. Rotation Method: Varimax with Kaiser normalization. Rotation converged in 3 iterations. KMO=.929, Bartlett’s test of sphericity: χ²(45) = 1636,821, P < .001.*

**Appendix D – Associations between effects of biosecurity measures and job conditions**

**Table D1. “**Biosecurity measures currently required, in the long term, will lead to better animal welfare” by profession. *

|  |  | **Other** | **Farmer** | **Total** |
| --- | --- | --- | --- | --- |
| 1 - Strongly disagree | Count | 13 | 46 | 59 |
|  | Expected Count | 24,5 | 34,5 | 59 |
| 2 | Count | 23 | 20 | 43 |
|  | Expected Count | 17,9 | 25,1 | 43 |
| 3 | Count | 22 | 28 | 50 |
|  | Expected Count | 20,8 | 29,2 | 50 |
| 4 | Count | 26 | 27 | 53 |
|  | Expected Count | 22 | 31 | 53 |
| 5 - Strongly agree | Count | 12 | 14 | 26 |
|  | Expected Count | 10,8 | 15,2 | 26 |
| Total | Count | 96 | 135 | 231 |
|  | Expected Count | 96 | 135 | 231 |

** X^2^(1) = 13.356, P = .010*

**Table D2. “**Biosecurity measures currently required, in the long term, will lead to better animal health” by profession. *

|  |  | **Other** | **Farmer** | **Total** |
| --- | --- | --- | --- | --- |
| 1 - Strongly disagree | Count | 13 | 46 | 59 |
|  | Expected Count | 24,5 | 34,5 | 59 |
| 2 | Count | 23 | 20 | 43 |
|  | Expected Count | 17,9 | 25,1 | 43 |
| 3 | Count | 22 | 28 | 50 |
|  | Expected Count | 20,8 | 29,2 | 50 |
| 4 | Count | 26 | 27 | 53 |
|  | Expected Count | 22 | 31 | 53 |
| 5 - Strongly agree | Count | 12 | 14 | 26 |
|  | Expected Count | 10,8 | 15,2 | 26 |
| Total | Count | 96 | 135 | 231 |
|  | Expected Count | 96 | 135 | 231 |

** X^2^(1) = 12.493, P = .014 **

**Table D3. “**Biosecurity measures currently required, in the short term, will lead to better animal welfare” by profession. *

|  |  | **Other** | **Farmer** | **Total** |
| --- | --- | --- | --- | --- |
| 1 - Strongly disagree | Count | 16 | 48 | 64 |
|  | Expected Count | 26,6 | 37,4 | 64 |
| 2 | Count | 18 | 17 | 35 |
|  | Expected Count | 14,5 | 20,5 | 35 |
| 3 | Count | 33 | 33 | 66 |
|  | Expected Count | 27,4 | 38,6 | 66 |
| 4 | Count | 20 | 24 | 44 |
|  | Expected Count | 18,3 | 25,7 | 44 |
| 5 - Strongly agree | Count | 9 | 13 | 22 |
|  | Expected Count | 9,1 | 12,9 | 22 |
| Total | Count | 96 | 135 | 231 |
|  | Expected Count | 96 | 135 | 231 |

** X^2^(1) = 10.844, P = .028 **

**Table D4. “**Biosecurity measures currently required, in the short term, will lead to better animal health” by profession. *

|  |  | **Other** | **Farmer** | **Total** |
| --- | --- | --- | --- | --- |
| 1 - Strongly disagree | Count | 11 | 38 | 49 |
|  | Expected Count | 20,4 | 28,6 | 49 |
| 2 | Count | 18 | 23 | 41 |
|  | Expected Count | 17 | 24 | 41 |
| 3 | Count | 30 | 30 | 60 |
|  | Expected Count | 24,9 | 35,1 | 60 |
| 4 | Count | 26 | 32 | 58 |
|  | Expected Count | 24,1 | 33,9 | 58 |
| 5 - Strongly agree | Count | 11 | 12 | 23 |
|  | Expected Count | 9,6 | 13,4 | 23 |
| Total | Count | 96 | 135 | 231 |
|  | Expected Count | 96 | 135 | 231 |

** X^2^(1) = 9.848, P = .043 **

**Table D5. “**Biosecurity measures currently required, have already led to better animal health” by profession. *

|  |  | **Other** | **Farmer** | **Total** |
| --- | --- | --- | --- | --- |
| 1 - Strongly disagree | Count | 12 | 46 | 58 |
|  | Expected Count | 24,1 | 33,9 | 58 |
| 2 | Count | 16 | 20 | 36 |
|  | Expected Count | 15 | 21 | 36 |
| 3 | Count | 29 | 27 | 56 |
|  | Expected Count | 23,3 | 32,7 | 56 |
| 4 | Count | 25 | 24 | 49 |
|  | Expected Count | 20,4 | 28,6 | 49 |
| 5 - Strongly agree | Count | 14 | 18 | 32 |
|  | Expected Count | 13,3 | 18,7 | 32 |
| Total | Count | 96 | 135 | 231 |
|  | Expected Count | 96 | 135 | 231 |

** X^2^(1) = 14.805, P = .005 ***

**Table D6. “**Biosecurity measures currently required have already led to better animal welfare” by the profession. *

|  |  | **Other** | **Farmer** | **Total** |
| --- | --- | --- | --- | --- |
| 1 - Strongly disagree | Count | 17 | 46 | 63 |
|  | Expected Count | 26,2 | 36,8 | 63 |
| 2 | Count | 16 | 26 | 42 |
|  | Expected Count | 17,5 | 24,5 | 42 |
| 3 | Count | 24 | 22 | 46 |
|  | Expected Count | 19,1 | 26,9 | 46 |
| 4 | Count | 24 | 24 | 48 |
|  | Expected Count | 19,9 | 28,1 | 48 |
| 5 - Strongly agree | Count | 15 | 17 | 32 |
|  | Expected Count | 13,3 | 18,7 | 32 |
| Total | Count | 96 | 135 | 231 |
|  | Expected Count | 96 | 135 | 231 |

** X^2^(1) = 9.632, P = .047 **

**Table D7. “**Biosecurity measures currently required lead to better animal health” by profession. *

|  |  | **Other** | **Farmer** | **Total** |
| --- | --- | --- | --- | --- |
| 1 - Strongly disagree | Count | 10 | 37 | 47 |
|  | Expected Count | 19,5 | 27,5 | 47 |
| 2 | Count | 16 | 23 | 39 |
|  | Expected Count | 16,2 | 22,8 | 39 |
| 3 | Count | 24 | 27 | 51 |
|  | Expected Count | 21,2 | 29,8 | 51 |
| 4 | Count | 33 | 34 | 67 |
|  | Expected Count | 27,8 | 39,2 | 67 |
| 5 - Strongly agree | Count | 13 | 14 | 27 |
|  | Expected Count | 11,2 | 15,8 | 27 |
| Total | Count | 96 | 135 | 231 |
|  | Expected Count | 96 | 135 | 231 |

** X^2^(1) = 10.717, P = .030 **

**Table D8. “**Biosecurity measures currently required lead to better animal welfare” by profession. *

|  |  | **Other** | **Farmer** | **Total** |
| --- | --- | --- | --- | --- |
| 1 - Strongly disagree | Count | 13 | 45 | 58 |
|  | Expected Count | 24,1 | 33,9 | 58 |
| 2 | Count | 20 | 17 | 37 |
|  | Expected Count | 15,4 | 21,6 | 37 |
| 3 | Count | 23 | 27 | 50 |
|  | Expected Count | 20,8 | 29,2 | 50 |
| 4 | Count | 25 | 31 | 56 |
|  | Expected Count | 23,3 | 32,7 | 56 |
| 5 - Strongly agree | Count | 15 | 15 | 30 |
|  | Expected Count | 12,5 | 17,5 | 30 |
| Total | Count | 96 | 135 | 231 |
|  | Expected Count | 96 | 135 | 231 |

** X^2^(1) = 12.637, P = .013 **
